# Transcriptomic Biomarkers to Discriminate Bacterial from Nonbacterial Infection in Adults Hospitalized with Respiratory Illness

**DOI:** 10.1038/s41598-017-06738-3

**Published:** 2017-07-26

**Authors:** Soumyaroop Bhattacharya, Alex F. Rosenberg, Derick R. Peterson, Katherine Grzesik, Andrea M. Baran, John M. Ashton, Steven R. Gill, Anthony M. Corbett, Jeanne Holden-Wiltse, David J. Topham, Edward E. Walsh, Thomas J. Mariani, Ann R. Falsey

**Affiliations:** 1 Division of Neonatology and Pediatric Molecular and Personalized Medicine Program, Department of Pediatrics, University of Rochester School Medicine, Rochester, NY USA; 2 Division of Allergy Immunology & Rheumatology, Department of Medicine, University of Rochester School Medicine, Rochester, NY USA; 3 Department of Biostatistics and Computational Biology, University of Rochester School Medicine, Rochester, NY USA; 4 Genomics Research Center, University of Rochester School Medicine, Rochester, NY USA; 5 David H. Smith Center for Vaccine Biology and Immunology, University of Rochester School Medicine, Rochester, NY USA; 6 Department of Microbiology and Immunology, University of Rochester School Medicine, Rochester, NY USA; 7 Division of Infectious Diseases, Department of Medicine, University of Rochester School Medicine and Rochester General Hospital, Rochester, NY USA

## Abstract

Lower respiratory tract infection (LRTI) commonly causes hospitalization in adults. Because bacterial diagnostic tests are not accurate, antibiotics are frequently prescribed. Peripheral blood gene expression to identify subjects with bacterial infection is a promising strategy. We evaluated whole blood profiling using RNASeq to discriminate infectious agents in adults with microbiologically defined LRTI. Hospitalized adults with LRTI symptoms were recruited. Clinical data and blood was collected, and comprehensive microbiologic testing performed. Gene expression was measured using RNASeq and qPCR. Genes discriminatory for bacterial infection were identified using the Bonferroni-corrected Wilcoxon test. Constrained logistic models to predict bacterial infection were fit using screened LASSO. We enrolled 94 subjects who were microbiologically classified; 53 as “non-bacterial” and 41 as “bacterial”. RNAseq and qPCR confirmed significant differences in mean expression for 10 genes previously identified as discriminatory for bacterial LRTI. A novel dimension reduction strategy selected three pathways (lymphocyte, α-linoleic acid metabolism, IGF regulation) including eleven genes as optimal markers for discriminating bacterial infection (naïve AUC = 0.94; nested CV-AUC = 0.86). Using these genes, we constructed a classifier for bacterial LRTI with 90% (79% CV) sensitivity and 83% (76% CV) specificity. This novel, pathway-based gene set displays promise as a method to distinguish bacterial from nonbacterial LRTI.

## Introduction

Acute respiratory infections (ARI) occur commonly throughout life, accounting for substantial morbidity and mortality in adults^1^. In most cases the precise microbial etiology is unknown and antibiotics are administered empirically in illness requiring hospitalization^2, 3^. Although sensitive molecular diagnostics such as polymerase chain reaction (PCR) allow clinicians to rapidly and accurately diagnose a wide variety of respiratory viruses, their impact on management and antibiotic prescription has been modest primarily due to concern about bacterial co-infection^4–6^. Such concerns are not unfounded as approximately 30% of hospitalized adults with viral LRTI have evidence of concomitant bacterial infection^7^. The study of bacterial lung infection has been hampered by insensitive tests for invasive disease and difficulty interpreting sputum cultures. Blood cultures are positive in only 6–10% of pneumonia cases and sputum is often contaminated with upper airway flora^8^. Clinical parameters such as fever, purulent sputum, white blood cell count and radiographic patterns provide insufficient precision to reliably distinguish viral from bacterial infections^9^. Thus, “ruling out” bacterial infection is extremely difficult, resulting in a default position of prescribing antibiotics to most patients hospitalized with LRTI. This results in significant antibiotic overuse, with resultant adverse effects and increased costs. Recently, serum biomarkers such as procalcitonin have shown some promise as a supplement to clinical judgment in assessing patients with LRTI but a need for more accurate tests remains^10^.

Gene expression profiling of peripheral blood mononuclear cells (PBMCs) or whole blood represents a powerful new approach for analysis of host responses during infection^11, 12^. Preliminary studies indicate viruses and bacteria trigger specific host transcriptional patterns, yielding unique “bio-signatures” that discriminate viral from bacterial infection^13–16^. Gene array analysis on extracted RNA from small volumes of blood from young children with febrile illnesses can differentiate infection with bacteria from viruses or virus plus bacteria, and also between Gram-positive and Gram-negative bacterial infection^14^. In a recent collaboration with Suarez, we identified ten classifier genes in adults hospitalized with LRTI that discriminated between bacterial and viral infection^15^. Eight of the ten were interferon (IFN) related genes that were over expressed in viral infection and absent in bacterial infection. The goal of the present study was to prospectively validate these, and discover additional classifier genes useful for discriminating bacterial from viral LRTI in hospitalized adults.

## Methods

### Population

Adults 21 years or older admitted to Rochester General Hospital (RGH), Rochester, New York, with diagnoses or symptoms compatible with acute LRTI from January through June 2013 using the same criteria as Suarez^15^. Admission logs were screened daily for patients with diagnoses of acute exacerbation of chronic obstructive pulmonary disease (AECOPD), bronchitis, asthma, influenza, viral syndrome, respiratory failure and congestive heart failure with infection, pneumonia or symptoms of wheezing, dyspnea, cough, sputum production, nasal congestion, sore throat, hoarseness. Patients were enrolled within 24 hours of admission and demographic, clinical and laboratory information collected. Exclusion criteria included antibiotic treatment before admission, immunosuppression, cavitary lung disease, and witnessed aspiration. The University of Rochester and RGH institutional review boards approved the study and written informed consent was obtained from subjects or authorized representatives. All study procedures were performed in accordance with the institutional policies and guidelines and regulations pertaining to research involving human subjects.

### Microbiologic Methods

Nose and throat swabs (NTS), sputum, urine, and blood samples obtained at admission for bacterial and viral detection were processed at RGH clinical laboratories, as described^7, 15^. Briefly, single blood cultures positive for organisms consistent with skin flora (coagulase negative staphylococcus, Corynebacterium, alpha hemolytic streptococci, *Propionibacterium acnes*) were considered contaminants. Sputum cultures were considered positive if ≥2+ of a pathogenic bacterium grew from an adequate sample using standard criteria. Urine was assayed for *Streptococcus pneumoniae* antigen using Binax NOW (Binax, Inc, Scarborough, ME). NTS and sputum were tested using the real time multiplex PCR (FilmArray Respiratory Panel, Idaho Technologies, Inc, Salt Lake City, UT) for detection of 15 viruses and 3 atypical bacteria. Subjects were only included in the analysis who had a microbiologic diagnosis and had adequate diagnostic testing. Illness definitions are listed in Table 1.

**Table 1:** Microbiologic Classification Criteria

Microbiologic Classification	
Virus infection alone	NTS or sputum sample positive for any virus by one of the following: RT-PCR [all viruses], and all tests for bacteria were negative.
Bacterial infection alone	Negative viral diagnostic tests and any of the following: (1) positive blood culture meeting criteria for a pathogen, (2) positive culture for a respiratory pathogen from an adequate sputum sample, (3) a positive urinary antigen test for *Streptococcus pneumoniae* or *Legionella pneumophila*, or (4) positive PCR for *Mycoplasma pneumoniae*, *Chlamydophila pneumoniae* or *Bordetella pertussis*.
Viral-Bacterial Infection	Meets definition for bacterial infection and viral infection
**Adequate Microbiologic Assessment**	Subjects with fever were required to have blood cultures prior to antibiotics and those with productive cough were required to have an adequate sputum sample obtained within 24 hours of admission and ≤6 hours after administration of antibiotics. If these criteria were not met subjects could not be considered bacterial negative.
**Analysis Groups**	
Bacterial	Subjects with bacterial infection alone and those with mixed bacterial - viral infection
Non-bacterial	Viral infection alone

### Molecular Methods

Approximately 12 ml of whole blood was collected in Tempus™ Blood RNA Tube at enrollment. Following centrifugation, RNA was isolated from the pellet using the Tempus Spin RNA Isolation Kit. For 10 subjects blood was collected in CPT tubes and RNA isolated from spin-purified PBMCs using the RNeasy mini kit. Total RNA was processed for globin reduction using GLOBINclear Human Kit.

For quantitative PCR, cDNA was synthesized from 250 ng RNA using iScript cDNA synthesis kit and quantitative PCR performed as described^16^ using noncommercial assays, Supplemental Table 1 (http://pga.mgh.harvard.edu/primerbank). Difference in gene expression was tested by Wilcoxon Rank test (p < 0.05).

For RNAseq, cDNA libraries were generated using 200 ng of globin-reduced total RNA from each sample. Library construction was performed using the TruSeq Stranded mRNA library kit (Illumina, San Diego, CA). cDNA quantity was determined with the Qubit Flourometer (Life Technologies, Grand Island, NY) and quality assessed using the Agilent Bioanalyzer 2100 (Santa Clara, CA). Libraries were sequenced (single end reads) on the Illumina HiSeq 2500 (Illumina, San Diego, CA) to generate 20 million reads/sample.

Reads were aligned using the TopHat algorithm and expression values summarized using HTSeq^17, 18^. Raw counts were normalized using Conditional Median normalization. Differences in expression between bacterial and non-bacterial infected subjects for each gene were assessed by Wilcoxon rank test at an FDR q < 0.05.

### Statistical Methods

LASSO-penalized logistic regression was used to select pathway-based genetic predictors of bacterial infection (Supplemental Table 2). Model parameters were selected via cross-validation (CV), and the model’s predictive ability was assessed by a nested cross-validated (NCV) estimate of the area under the ROC curve (AUC). Briefly, genes were univariately screened, and those with a nominal Wilcoxon p < 0.10 were assigned to 1330 known canonical pathways, as defined in the Molecular Signature Database (MSigDB) of Broad Institute^19^. The first principal component (PC1) of the genes in each pathway was derived, and genes with loadings close to 0 were removed. Pathways were subjected to an additional univariate Wilcoxon screen with significance level selected by CV. LASSO was then applied to these pathway PC1s to obtain a logistic model with pathway predictors.

Clinical variables were dichotomized for robustness and ease of interpretation, univariately screened using nominal α = 0.05, and all-subsets selection was used to build a clinical-only logistic regression model with the number of variables selected via CV.

### Data Availability

DbGAP accession code : phs001248.v1.p1

## Results

### Demographics and Illness Characteristics

Two hundred and thirteen patients were enrolled; 100 had definitive microbiologic diagnoses and 94 generated transcriptomic data passing quality control metrics. Clinical characteristics of these 94 subjects are shown in Table 2. Mean age was 61 years, 55% were female and 78% were Caucasian. The majority (98%) had at least one chronic medical condition, 7% required intensive care and none died. Clinical diagnoses included asthma exacerbation (n = 17), bronchitis (n = 25), AECOPD (n = 21), pneumonia (n = 23) and bacteremia (n = 8). For molecular analyses, 41 subjects were considered “bacterial” infection (27 bacterial only and 14 mixed viral/bacterial) and 53 subjects were classified “non-bacterial” infection (viral infection alone). A wide range of pathogens were detected (Supplemental Table 3) with influenza A the most common virus and *Streptococcus pneumoniae* the most common bacteria documented.

**Table 2:** Subject Demographic and Clinical Characteristics

	**All** N = 94	**Bacterial** (Bacterial alone and Mixed Bacterial Viral) N = 41	**Non Bacterial** (Viral alone) N = 53	Fisher’s Exact or t-test P value
**Demographics**				
Age, mean ± SD	61 ± 18	67 ± 18	57 ± 17	0.01
Male Sex, No. (%)	42 (45)	21 (51)	21 (40)	0.30
White Race	73 (78)	35 (85)	38 (72)	0.14
**Underlying Conditions**				
COPD, No. (%)	30 (32)	17 (41)	13 (25)	0.12
CHF, No. (%)	23 (24)	10 (24)	13 (25)	1
Diabetes, No. (%)	27 (29)	13 (32)	14 (26)	0.65
**Symptoms**				
Nasal Congestion, No. (%)	50 (53)	14 (34)	36 (68)	0.002
Cough, No. (%)	85 (90)	34 (83)	51 (96)	0.04
Sputum, No. (%)	66 (70)	29 (71)	37 (70)	1
Dyspnea, No. (%)	81 (86)	34 (83)	47 (89)	0.55
Rigors, No. (%)	30 (32)	12 (29)	18 (34)	0.66
**Physical Findings**				
Wheezing, No. (%)	66 (70)	25 (61)	41 (77)	0.11
Rales, No. (%)	29 (31)	15 (37)	14 (26)	0.37
Temperature (°C)	37.8 ± 1.0	37.8 ± 0.9	37.8 ± 1.0	0.93
Systolic Blood Pressure, mean ± SD	112 ± 21	111 ± 25	113 ± 18	0.73
Oxygen Saturation, mean ± SD	90.0 ± 6.0	88.4 ± 7.1	91.2 ± 4.7	0.03
**Laboratory Data**				
Infiltrate on Chest Radiograph	27 (29)	21 (52)	6 (11)	<0.0001
White blood cell count, mean ± SD	10.7 ± 5.6	13.7 ± 6.8	8.5 ± 2.9	<0.0001
% bands in peripheral blood, mean ± SD	3.1 ± 5.6	5.2 ± 6.9	0.7 ± 1.9	0.002
Blood urea nitrogen, mean ± SD	19 ± 12	23 ± 15	16 ± 8	0.005

### Transcriptomic Data Quality

Six of the 100 samples were excluded based on poor RNA or sequence quality (read count/mapped read numbers) or if they were outliers in unsupervised cluster analysis, leaving 94 samples for analysis. On average 38 ± 5.6 million reads were generated from each of the cDNA libraries using globin-reduced, unfractionated whole blood RNA, with a mapping rate of 89.8 ± 2.8% indicating high quality sequence data. Genomic coverage averaged 66.1 ± 6.5% of the human transcriptome (Supplemental Figures S1A, S1B) indicating appropriate diversity of transcript sampling. Of 25,559 mapped genes, 2,440 genes with zero counts across all subjects were excluded. In addition, 7,438 genes with normalized counts of <3 for >75% subjects were excluded leaving 15,681 genes for analysis (Supplemental Figure S1C). RNA concentration was similar for blood collected in CPT and Tempus tubes (Supplemental Figure S1D).

### Replication of Array-Based Predictors

**Figure 1:**
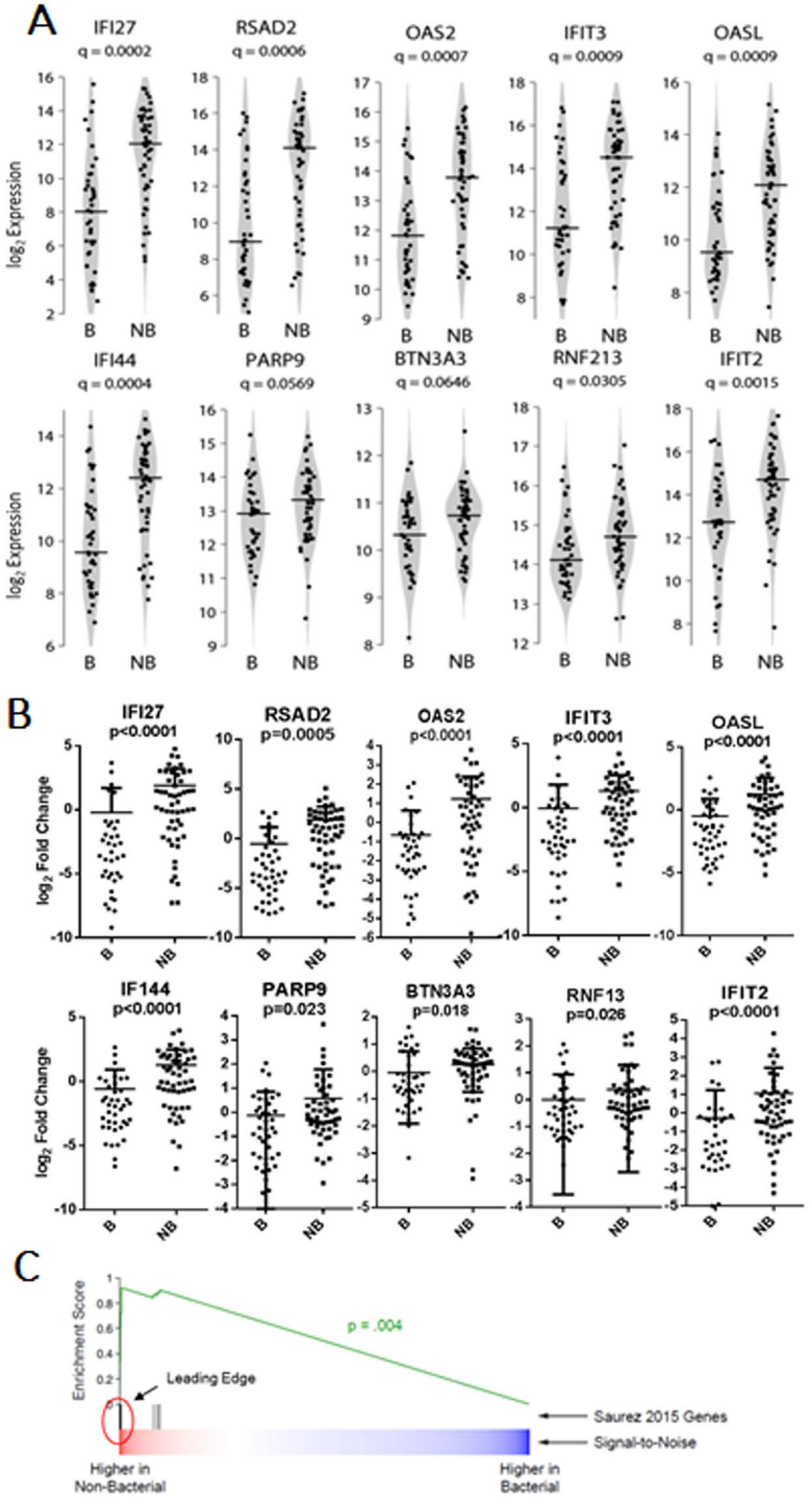
Replication of Array-Based Predictors. (**A**) Violin plots of RNASeq-based expression data for each of the 10 genes. Q-values for differential expression between bacterial and non-bacterial groups are indicated beneath the gene names. Horizontal lines indicate group medians. (**B**) Quantitative reverse transcriptase-polymerase chain reaction (qPCR)- based expression data for each of the 10 genes. P-values for differential expression between bacterial and non-bacterial groups are indicated beneath the gene names. Horizontal lines indicate group medians. (**C**) Gene set enrichment analysis plot for these 10 genes. Seven genes (vertical black lines) are contained in the leading edge of the enrichment plot.

The expression of ten marker genes, previously identified by Suarez^15^, were assessed using RNA-Seq and qPCR for the ability to distinguish bacterial from non-bacterial illness. In the transcriptomic data, 8 of the 10 genes demonstrated significant differences between groups using Wilcoxon Rank test at a False Discovery Rate (FDR) or q < 0.05 (Fig. 1A). By qPCR all ten showed significant difference between bacterial and non-bacterial groups by Wilcoxon Rank test at a nominal p-value < 0.05 (Fig. 1B). Of note, increased expression of all 10 genes is associated with non-bacterial infection, with most belonging to the interferon family (Fig. 1C). Gene Set Enrichment Analysis (GSEA)^20^ using those ten genes as a gene set provided high enrichment scores (ES) from seven of those genes (IFI27, RSAD2, IFI44, IFIT3, OASL, OAS2, and IFIT2) in the leading edge of the plot indicating these genes are relatively informative for distinguishing groups.

### Novel Markers for Bacterial Infection

**Figure 2:**
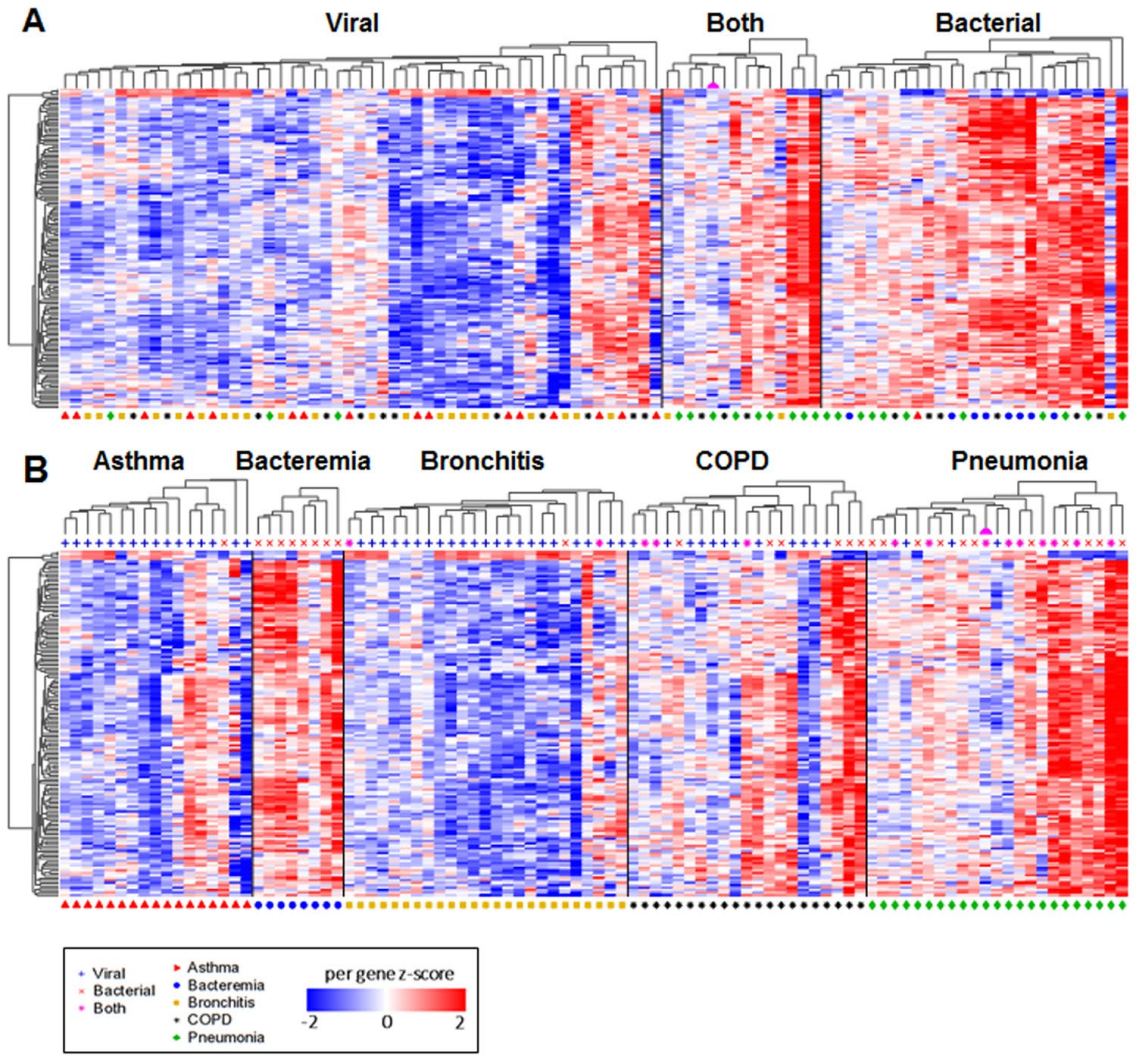
Global Expression Patterns in LRTI. (**A**) Shown is a heat map for the 141 genes (rows) demonstrating significantly different expression levels between bacterial and non-bacterial groups. Each column represents an individual subject, and subjects were grouped based upon microbiological diagnosis, using independent hierarchical clustering (Euclidean distance with average linkage). (**B**) The same data are presented, grouped by clinical diagnosis. The pink semi-circle denotes the patient with influenza A and a single blood culture positive for *S*. *aureus*.

In addition to validating these previously identified markers for bacterial infection^15^, we sought to identify novel gene sets with the greatest potential for accurate classification. Using the Wilcoxon rank test with FDR q < 0.05, we identified 141 genes that are differentially expressed between subjects with bacterial versus non-bacterial infections (displayed in Figs. 2A and B). In contrast to the predictive markers replicated above, most of these 141 genes have higher expression in most subjects with bacterial infection. Notably, reduced expression of these 141 genes was observed in patients with a clinical diagnosis of asthma and bronchitis, whereas patients with pneumonia and bacteremia tended to have increased expression of these genes, with AECOPD presenting a mixed pattern. A subset of nine genes was selected for molecular validation by qPCR, based on their magnitude of difference or biological relevance (Supplemental Figure S2A). We validated significant differences in expression for 8 of the 9 genes (Supplemental Figure S2B).

Ingenuity Pathway Analysis (IPA) was used to define the biology represented by genes differentially expressed between nonbacterial vs. bacterial infections (Supplemental Figures S3A, S3B and S3C). For this we analyzed various differentially expressed gene sets, based upon q-value thresholds; from q < 0.01 (n = 1434) to q < 0.0005 (n = 304). Not surprisingly, this analysis predicted inhibition of numerous viral infection and replication related biological functions (Figure S3A). Pathways analysis also implicated virus related pathways (interferon signaling, activation of IRF), as well as non-viral pathways (Figure S3B). Of note, inhibition of integrin signaling, activation of RhoGDI signaling and involvement (no direction predicted) of the IGF1 signaling pathways were also predicted. Our analyses also sought to predict regulatory molecules that may drive differential expression distinguishing bacterial from non-bacterial infection subjects (Figure S3C). This implicated multiple regulators of interferon signaling (IRF3, IRF9, STAT1/2). The most significant regulator identified was CNOT7, reported to be responsible for dampening interferon signaling through STAT1^21^.

### Assessment of Gene Expression Markers for Bacterial LRTI

**Figure 3:**
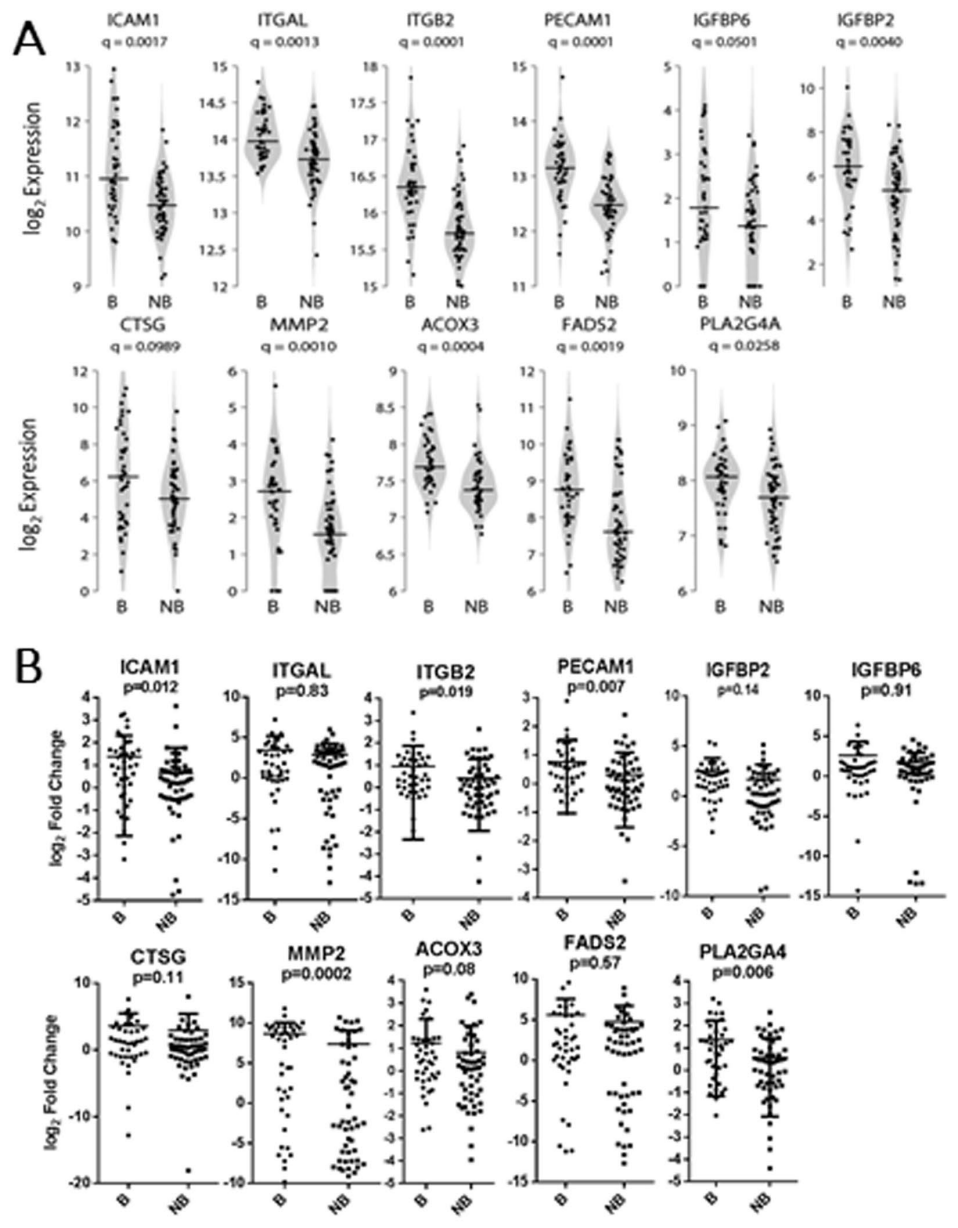
Expression of Genes Predictive for Bacterial Involvement in LRTI. (**A)** Violin plots of RNASeq-based expression data for 11 selected genes, from 3 canonical pathways, that provide predictive value for identifying bacterial involvement in lower respiratory track infections. Q-values for differential expression between bacterial and non-bacterial groups are indicated beneath the gene names. Horizontal lines indicate group medians. (**B**) Quantitative reverse transcriptase-polymerase chain reaction (qPCR)- based expression data for each of the 11 genes. P-values for differential expression between bacterial and non-bacterial groups are indicated beneath the gene names. Horizontal lines indicate group medians.

**Figure 4:**
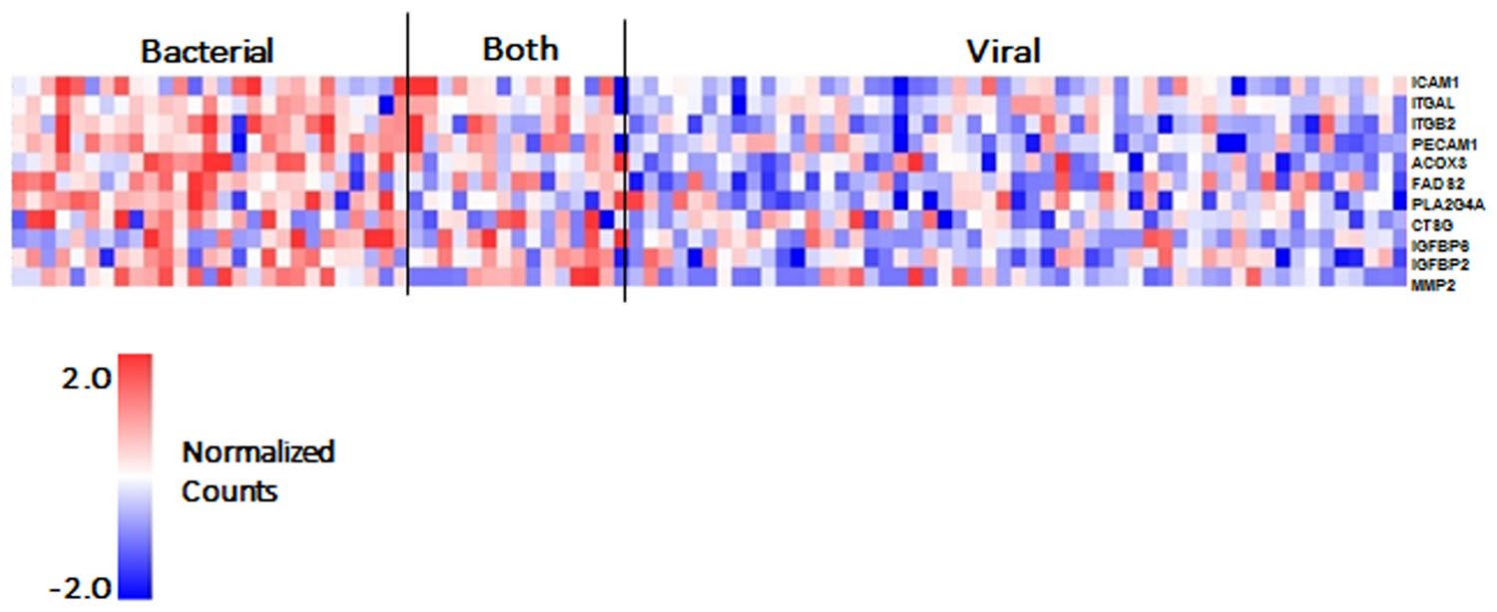
Shown is a heat map for the 10 predictive genes identified by pathway analysis as predictive of bacterial infection (rows) demonstrating differential expression in the 3 groups, bacterial, mixed viral bacterial and viral alone. Each column represents an individual subject.

**Figure 5:**
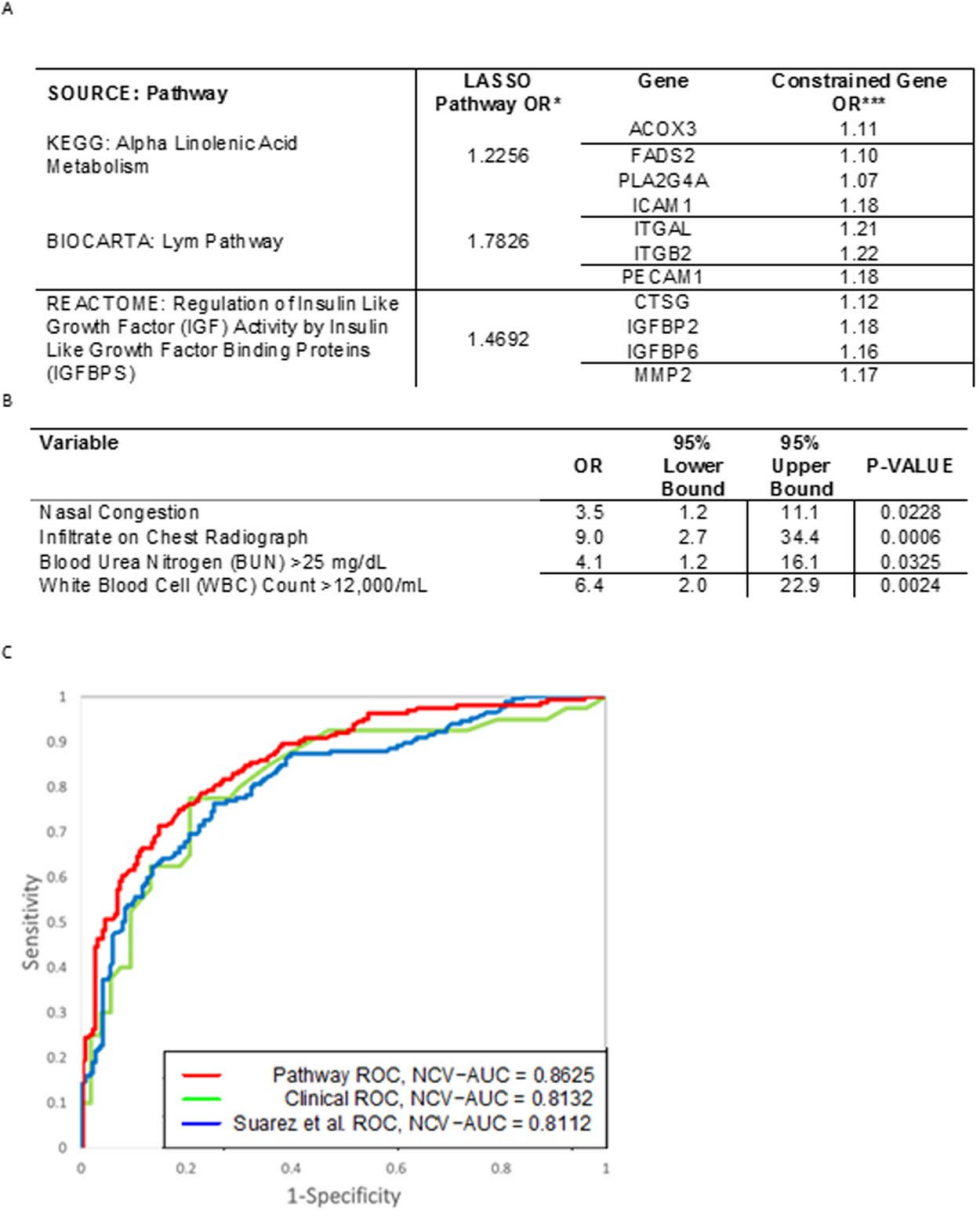
Comparison of Predictive Performance for Gene Expression and Clinical Biomarkers. (**A**) LASSO-penalized logistic regression retained 3 pathways consisting of a total of 11 genes providing the greatest predictive value for classifying subjects as bacterial or non-bacterial. *LASSO Pathway OR are odds ratios per SD of the hard-thresholded 1st PC of the nominally significant genes within the pathway. ***Constrained Gene OR = exp (Gene Loading * log (LASSO Pathway OR)/SDPathway) = (LASSO Pathway OR)(Gene Loading/(SD of Pathway)). (**B)** Cross-validation chose a 4-predictor model consisting of nasal congestion, infiltrates on chest radiograph, blood urea nitrogen levels and white blood cell count. (**C)** Area Under the ROC Curve (AUC) characteristics are shown for fully nested cross-validated estimates using the “pathway”-selected 11 gene set, the “array”-selected 10 gene set, and the 4 clinical variables. These data indicate that our pathway-based 11-gene predictor outperforms both the clinical and array-based gene models.

We identified a set of genes whose expression may be useful for classification of bacterial versus non-bacterial infections. We leveraged biological priors as a means of identifying the most robust predictors since^1^ expression changes at the individual gene level alone may not be sufficient to identify biologically meaningful data and^2^ there exist substantial statistical advantages to dimension reduction strategies in the analysis of genome-wide data. We used a curated list of genes^20^ to partition our transcriptomic data into 1330 biologically-relevant gene sets, or pathways. Gene expression data from the genes in each pathway were reduced to a single derived pathway variable as described in the Statistical Methods. Cross-validation simultaneously selected a LASSO penalty parameter and a Bonferroni-corrected significance level of 0.05 for screening pathways, at which 43 pathways were univariately associated with infection status. Of those, LASSO-penalized logistic regression retained 3 pathways consisting of a total of 11 genes (Fig. 3A) providing the greatest predictive value for classifying subjects as bacterial or non-bacterial. Differential expression was confirmed for 6 genes by qPCR (Fig. 3B). A Heat map demonstrating the differential expression of 11 selected genes between subjects with bacterial, mixed viral bacterial and viral infection alone is shown in Fig. 4. Pathway and gene names, pathway odds ratios (OR) based upon LASSO and constrained gene OR are presented in Fig. 5A. Sensitivity-specificity analysis indicated models using these gene sets provided a naive area under the receiver-operator curve (AUC) of 0.94, and a conservative, fully nested, cross-validated (NCV) AUC of 0.86.

Clinical variables were also tested for the ability to distinguish subjects with bacterial versus non-bacterial infection using the screened all subsets selection method described in the Statistical Methods. Cross-validation chose a 4-predictor model consisting of nasal congestion, infiltrates on chest radiograph, blood urea nitrogen levels and white blood cell count (Fig. 5B). This clinical model had a surprisingly robust naïve AUC of 0.833 and an NCV-AUC of 0.813 (Fig. 5C). We also attempted to optimize biomarker selection by combining clinical and gene expression data. All such models failed to select any clinical variables.

Finally, we fit a model using only the 10 genes previously identified by Suarez^15^. Using LASSO-penalized logistic regression, we identified a stable 5-gene model with an NCV-AUC of 0.811. Therefore, our novel pathway-based biomarkers out-performed both the set of previously implicated genes and clinical variables (Fig. 5C).

**Figure 6:**
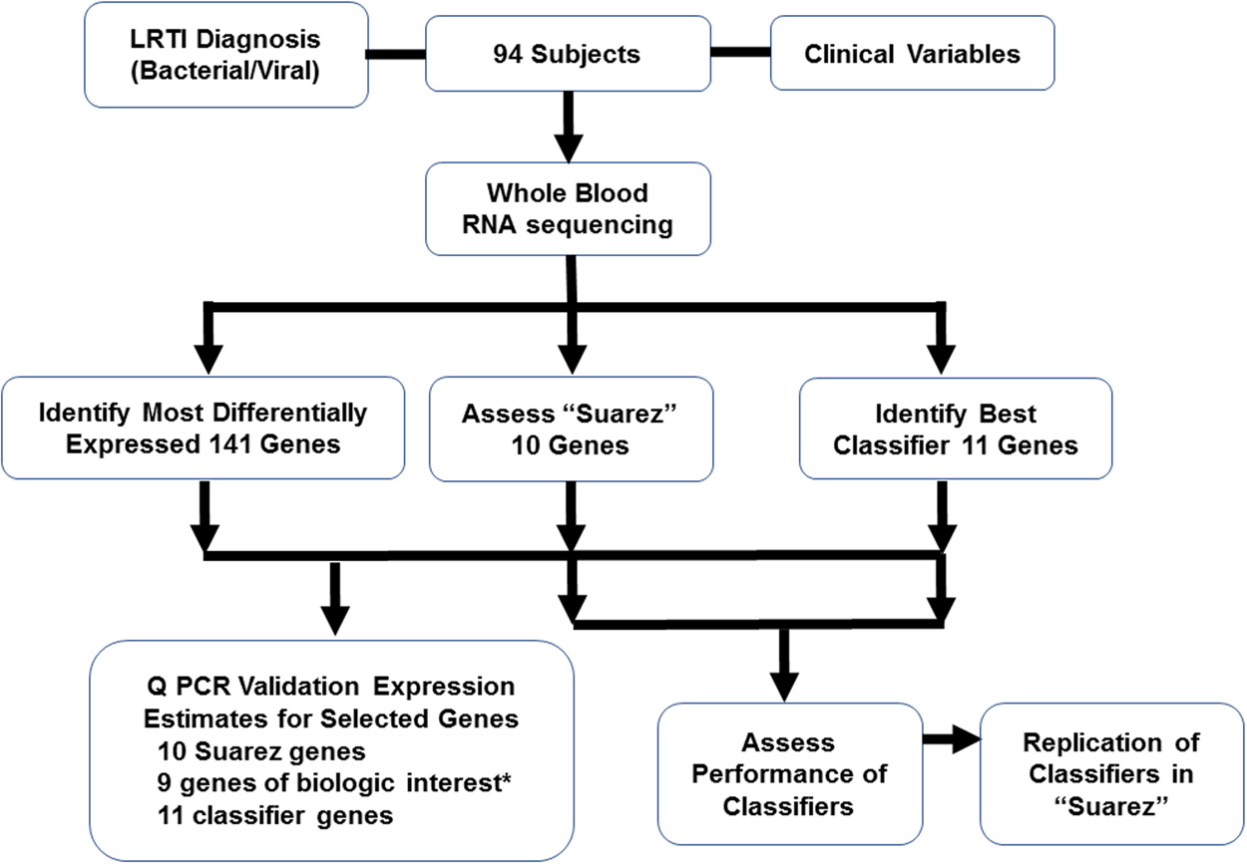
Flow chart of the analysis of different gene sets derived from RNA sequencing data from 94 subjects with confirmed bacterial-involved or non-bacterial LRTI selected for RNAseq interrogation. We first assessed expression of 10 genes identified by Suarez *et al*. to be differentially expressed comparing bacterial to viral infections. We next identified the 141 most differentially-expressed genes, as defined by statistical differences in bacterial vs. non-bacterial LRTI. Separately, we used a pathway-based approach to develop a novel gene expression classifier for discriminating bacterial vs. non-bacterial LRTI. *An additional 9 genes of biologic interest were selected from the 141gene set after considering the 10 Suarez genes. Validation of RNAseq-based expression estimates for selected genes, of particular biological interest, was attempted by qPCR. Finally, performance of the novel 3 pathway-based 11 gene classifier was assessed in our cohort.

To summarize the different analyses, a flow chart of the analysis of the different gene sets derived from RNA sequencing data from the 94 study subjects is provided in Fig. 6. We first assessed expression of 10 genes identified by Suarez *et al*. to be differentially expressed comparing bacterial to viral infections. We next identified the 141 most differentially-expressed genes, as defined by statistical differences in bacterial vs. non-bacterial infection. Separately, we used a pathway-based approach to develop a novel gene expression classifier for discriminating bacterial vs. non-bacterial infection. An additional 9 genes of biologic interest were selected from the 141gene set after considering the 10 Suarez genes. Validation of RNAseq-based expression estimates for selected genes was attempted by qPCR. Finally, performance of the novel 3 pathway-based 11 gene classifier was assessed in our cohort.

### A Molecular Classifier for Bacterial LRTI

To classify subjects as bacterial or non-bacterial, it is necessary to threshold a molecular predictor. We estimated the sensitivity and specificity associated with several candidate thresholds for the nominal predicted probability of a bacterial infection (Supplemental Table 4). We targeted a threshold with sensitivity ≥ specificity ≥ 70%, corresponding with weighting errors among the bacterial subjects 50% higher than those among the non-bacterial subjects. The optimal threshold was estimated to be about 0.40, with a naive sensitivity of 90% and specificity of 83%, and a NCV sensitivity of 79% and specificity of 76%.

A particularly challenging group of subjects are those with mixed viral bacterial infections. Of the 14 subjects with mixed infection 12 (naïve sensitivity, 85.7%) were correctly classified by our predictors which was similar to the bacterial infection only group, 27/29 (93.1%). Using a threshold of 0.36 for the clinical predictors and 0.42 for the Suarez genes to calculate naïve sensitivity we found that 12 of 14 (85.7%) were correctly categorized by the clinical variables, whereas, 8 of 14 (57.1%) were correctly classified by the model we built using the 10 genes selected by Suarez *et al*. There was one subject misclassified as non-bacterial by all 3 models, while all other misclassified subjects differed by model.

Clinical information on the naively misclassified subjects is provided in Supplemental Table 5. Of note, one subject with influenza A and one of two sets of blood cultures positive for methicillin resistant *Staphylococcus aureus* (MRSA) was classified as non-bacterial (indicated by the pink semi-circle on the heat map in Figs. 2A and B, and in Supplemental Figure S4 (showing only subjects with *Staphylococcus aureus* infections). Additional clinical information about this subject is provided in Figure S4.

## Discussion

Respiratory infections are common reasons for hospitalization in adults and although broad-spectrum antibiotics are frequently prescribed, this practice is now being questioned^2, 22^. While progress has been made in viral detection, the inaccessibility of the primary site of bacterial infection (the bronchi and lung) makes accurate bacterial diagnostics difficult to develop. Because blood samples can be obtained in most patients, identifying circulating biomarkers reflecting pathologic processes in the lower airways is highly desirable^6, 23, 24^. A variety of protein biomarkers including C-reactive protein and pro-calcitonin (PCT) have been used singly and in combination to discriminate bacterial from viral infection^24–26^. While PCT has been used with some success to guide antibiotic treatment for LRTI, threshold levels have never been validated with microbiology^10, 27^.

As an alternative to serum protein biomarkers, gene expression analyses using peripheral blood have been used in cancer, cardiovascular, autoimmune and infectious diseases to study disease pathogenesis, severity and recently as a diagnostic tool^11, 16, 28–30^. Microarrays have been used to determine unique host response expression “signatures” for tuberculosis, malaria, bacterial and viral infections^13–16, 31, 32^. These signatures have been used to differentiate viral from bacterial disease infection, symptomatic from asymptomatic viral infections and to identify specific bacterial and viral pathogens^29, 33^.

Several studies have evaluated gene expression by microarray for diagnostic purposes in adults and children with ARI and febrile illness. Interestingly, despite similar accuracy of predictive gene sets (AUC ranging from 78–94%), there has been little overlap in predictive genes identified^13–16, 33–37^. Diverse populations and control groups studied plus alternate analytic tools used likely explain the different predictive genes identified. Developing a model with the goal of “ruling out bacterial infection” such as ours might be expected to yield different results than those with a goal of identifying influenza regardless of bacterial status^34, 35, 38^. Recently, Tsalik used micro array to assess gene expression in whole blood to discriminate bacterial from viral infection or non-infectious cardiopulmonary illness in 273 subjects with community onset ARI^16^. These investigators used sparse logistic regression to define 130 predictor genes in a model with an accuracy of 87% to discriminate clinically adjudicated bacterial, viral, and non-infectious illness.

One of the goals of our study was to prospectively validate the 10 predictor genes identified by Suarez in an independent cohort of hospitalized adults^15^. Despite the relatively small sample size, we confirmed differential expression of all 10 genes. In addition, we included additional discovery efforts since RNAseq has not previously been used for diagnostic purposes in LRTI. We discovered a number of new differentially expressed genes. Interestingly, in contrast to prior studies which detected genes increased in non-bacterial LRTI, most of our novel genes show increased expression in bacterial LRTI. Of note, subjects were enrolled at the same community hospital and infection status determined using the same criteria as in the Suarez study. In addition to technical differences in microarray and RNAseq methods, predicting bacterial vs. non-bacterial rather than distinguishing viral, bacterial and mixed viral/bacterial infections may have reduced the prominence of interferon related genes identified in the current study. Regardless, our methods for interpretation of differentially expressed genes (Figure S3) clearly predict the involvement of viral responses and activation of interferon signaling.

The use of clinical variables, even in combination with laboratory variables, has not successfully discriminated bacterial from viral infection with sufficient precision to be useful^9^. Although the predictive accuracy of our clinical variable model was almost as high as our model using gene expression data, this is likely explained by over representation of “extreme phenotypes”. To avoid misclassification, patients with bacteremic pneumonia and virally induced asthma exacerbations represented a substantial proportion of the subjects in our study. Although necessary when developing predictor gene sets, these groups are not the most troublesome groups for clinicians. Non-bacteremic pneumonia and AECOPD are frequently associated with diagnostic uncertainty and present more difficult antibiotic management decisions. Given the difficulty in establishing microbiologic diagnoses, studies with larger samples sizes are needed to evaluate if predictive genes identified in the current study can be applied to these populations. In addition, it may be useful to evaluate pathway-based gene sets with selected clinical variables to achieve optimal diagnostic accuracy (although this did not improve prediction in the current study).

In order for gene expression analysis to move from bench to clinic, a limited number of optimal predictive genes must be identified for which rapid PCR can be performed, and thresholds must be chosen to categorize patients as bacterial or non-bacterial. The molecular classifier we report here displays good sensitivity and specificity for predicting bacterial involvement in LRTI, but there is clearly room for improvement otherwise such predictors will not be adopted for clinical use. Unlike other groups, we undertook the challenging task of classifying mixed viral bacterial infections as bacterial. Since many clinicians currently use PCR testing to diagnose viral infections but prescribe antibiotics despite positive results because of fear of bacterial co-infections the ability to correctly categorize this group is important^4, 7^. No test will be perfect and sacrificing specificity to optimize sensitivity may be the most viable approach since the current default position is to prescribe antibiotics to hospitalized patients with respiratory infection. One patient in our study with influenza A pneumonia and a single blood culture positive for MRSA was classified as non-bacterial using our pathway-based gene model. In clinical practice blood isolates of *S*. *aureus* are almost never dismissed as a contaminant although the organism is a skin commensal. Interestingly, the gene expression pattern of this patient is markedly different than other patients with *S*. *aureus* bacteremia raising the possibility that the blood culture was a contaminant (Fig. 2B and Supplemental Figure S4, Clinical data provided in supplement).

Because the current work was designed as a validation and discovery study, the primary limitation is the small sample size. Thus, we chose to use all available data to build the best final model, rather than setting aside a substantial portion as a validation data set. However, cross-validation was used to avoid over-fitting the training data. Moreover, in a rigorous and computationally intensive move, an outer loop of nested cross-validation was used as an unbiased method to evaluate overall performance of the internally cross-validated model fitting procedure. Compared with data splitting, nested cross-validation results in better models and more stable estimates of performance, since every subject contributes to the performance estimates. The trade-off is that estimated performance does not correspond directly to the final model but rather to an average of several models fit to different data subsamples. Additionally, a larger sample size would allow sub-analyses of various microbiologic and clinical syndromes such as AECOPD and pneumonia, which may yield different optimal predictive gene sets. We considered validating our results utilizing publicly available data from similar subjects; however, there are concerns that inherent differences in methodology (RNAseq vs. gene array) and differences in subject demographics, population age, and rigor of microbiological classification may invalidate such comparisons. As additional similar data sets are added to the literature, meta-analysis might prove useful in identifying robust gene sets for discriminatory purposes. Finally, we did not include ill subjects with noninfectious conditions and thus we cannot conclude that our predictors are specific for bacterial infection. Although our predictors discriminate bacterial and nonbacterial illnesses this may reflect severity of illness rather than a bacterial specific signature. Strengths of the current study are the use of high throughput sequencing to classify bacterial and non-bacterial subjects, representing a novel unbiased diagnostic approach as well as inclusion of mixed viral bacterial infections in the predictive model.

In conclusion, this report adds to mounting evidence that gene expression analysis of peripheral blood can be a useful test to discriminate bacterial and nonbacterial respiratory illness in hospitalized adults. Additional prospective studies are needed to define optimal predictive genes, with or without clinical variables and serum biomarkers, to assist clinicians in limiting unnecessary antibiotics for respiratory infections.

## Electronic supplementary material

Supplementary Information
