# Supplementary material for: Transcriptomic Biomarkers to Discriminate Bacterial from Nonbacterial Infection in Adults Hospitalized with Respiratory Illness
Source: Sci Rep. 2017 Jul 26;7:6548. doi: 10.1038/s41598-017-06738-3 (PMC5529430; doi:10.1038/s41598-017-06738-3)

**<u>Data Supplement for:</u>**

# Transcriptomic Biomarkers to Discriminate Bacterial from Nonbacterial Infection in Adults Hospitalized with Respiratory Illness

Soumyaroop Bhattacharya*[1], Alex F Rosenberg*[2], Derick R Peterson*[3], Katherine Grzesik[3], Andrea M Baran[3], John M Ashton[4], Steven R Gill[4], Anthony M Corbett[3], Jeanne Holden-Wiltse[3], David J. Topham [5,6], Edward E Walsh[7], Thomas J Mariani[1], Ann R Falsey[7]

[1]Division of Neonatology and Pediatric Molecular and Personalized Medicine Program, Department of Pediatrics, [2]Division of Allergy Immunology & Rheumatology, Department of Medicine, [3]Department of Biostatistics and Computational Biology, [4]Genomics Research Center, [5]David H. Smith Center for Vaccine Biology and Immunology, [6] Department of Microbiology and Immunology and [7]Division of Infectious Diseases, Department of Medicine University of Rochester School Medicine and Rochester General Hospital, Rochester, NY, USA

*These authors made equal contributions to the work described.

## Supplemental Figure Legends

### Figure S1 Transcriptomics data quality metrics

Shown here are total number of input reads (A), percentage of input reads mapped to the genome (B), and the proportion of the genome for which transcripts were detected (C), for each individual sample (D) Shown here are the RNA concentrations of blood collected in Tempus tubes (circles) and CPT tubes (squares)

### Figure S2 Novel Expression Biomarkers for Bacterial Infection.

***A,*** Violin plots of RNASeq-based expression data for 9 selected genes with differential expression between bacterial and non-bacterial groups. Q-values for differential expression between bacterial and non-bacterial groups are indicated beneath the gene names. Horizontal

lines indicate group medians. ***B,*** Quantitative reverse transcriptase-polymerase chain reaction (qPCR)- based expression data for each of the 9 genes. P-values for differential expression between bacterial and non-bacterial groups are indicated beneath the gene names. Horizontal lines indicate group medians.

**Figure S3. Functional and Pathway Assessment of Novel Expression Biomarkers.**

Ingenuity Pathway Analysis (IPA) was used to identify enriched biological functions (**A**) and canonical pathways (**B**) represented by the 141 genes demonstrating significantly different expression levels between bacterial and non-bacterial groups, or subgroups of these genes, as described. The gene sets are: q < .01: n = 1434; q < .005: n = 1051; q < .001: n = 433; q < .0005: n = 304; q < .01, up-regulated only: n = 85. Gene sets are listed in columns, functions/pathways are listed in rows, and significance is displayed as the $-\log_{10}$ Benjamini-Hochberg (B-H) corrected p-value. Orange/blue circles indicate increased/decreased function, respectively, based on the activation z-score which measures the degree to which coordinate changes of the function's constituent genes are consistent with its activity. ***C,*** IPA was also used to statistically identify regulatory molecules capable of coordinating regulation of the 85 genes demonstrating significantly increased expression levels in the bacterial group. Predicted upstream regulators are shown where the –log10 of the overlap p-value was > 2.5. Bars are colored orange/blue if data suggests increased/decreased activity of the regulatory molecule.

**Figure S4. Global expression patterns in LRTI restricted to patients with Staphylococcal bacteremia.**

Shown is a heat map for the 141 genes (rows) demonstrating significantly different expression levels between bacterial and non-bacterial groups. Each column represents an individual subject with the orange Xs at top of columns indicating patients with bacterial classification and the pink * indicating viral-bacterial classification (influenza A and methicillin resistant staphylococcal aureus) The black semi-circle at the top denotes the patient misclassified by the predictor genes as nonbacterial.

Case Description: This patient was a 73 year-old woman with a history of oxygen dependent COPD. She had been hospitalized 6 days' prior with productive cough, wheezing and difficulty breathing. She had a negative influenza PCR at that time and adequate sputum that showed gram negative diplococci on Gram stain and grew 4+ Haemophilus influenzae and was discharged on moxifloxacin and a steroid taper for presumed AECOPD. She returned to the hospital complaining of increased difficulty breathing with wheezing and a dry cough. On exam, she had a normal blood pressure with temperature of 37.4 and was noted to have diffuse wheezing and respiratory distress. Oxygen saturation was 88% on 2 liters of oxygen and chest radiograph showed a possible infiltrate in the left lower lobe. Peripheral white blood cell count was 16,800 with 2% bands and 2% atypical lymphocytes. Nasal swab was PCR positive for influenza A H1N1 and one set of blood cultures on admission grew MRSA. Oseltamivir, Cefepime and flagyl were administered for possible healthcare associated pneumonia. Two additional sets of blood cultures drawn prior to receipt of vancomycin were negative. She was treated with 2 weeks of IV vancomycin and prednisone and gradually improved.

**Supplemental Table 1.** Primers for qPCR for selected genes

| Gene | Forward Primer | Reverse Primer |
|---|---|---|
| SIGLEC10 | AGATTCTACCGAAGAGACGGAC | CGTCGGGACCACATTGATGTA |
| LTA4H | ATGAGTGCTATTCGTGATGGAGA | TGGGCCAATTTGCCTGCTT |
| AATF | CCAGGGTGATTGACAGGTTTG | CCAGTTTTCTAATGCTACCCACT |
| TNFAIP8L3 | GATTCTGAGCAAAATAGCCAGCA | GGCTTCCTTCTTGTTGTGTGT |
| SIGLEC1 | CCACTAGGGCTGATACTGGCT | GAGGCGGGTGGTTGACTAC |
| C11orf82 | GTTGTCCACCTTCGTTACTCAG | GAGAATGGCAGATCATCCCAAAT |
| FAM101B | AGTGGAGTTTGACCCCTTACC | GAAGTGCCTCTCGGAGTCGTA |
| PPM1N | CGAGCGTTGGGCGACTTTA | CAGGAGCATGAACTCGTCCTC |
| PCOLCE2 | TACTTGGAAAATCACAGTTCCCG | CGGCACAGGTTGTCACTCTC |
| AGRN | GTCCTGCGTCTGCAAGAAGAG | CTCGCATTCGTTGCTGTAGG |
| IFI27 | TGCTCTCACCTCATCAGCAGT | CACAACTCCTCCAATCACAACT |
| RSAD2 | TTGGACATTCTCGCTATCTCCT | AGTGCTTTGATCTGTTCCGTC |
| OAS2 | CTCAGAAGCTGGGTTGGTTTAT | TTTATCGAGGATGTCACGTTGG |
| IFIT3 | AGAAAAGGTGACCTAGACAAAGC | CCTTGTAGCAGCACCCAATCT |
| IFI44 | GGTGGGCACTAATACAACTGG | CACACAGAATAAACGGCAGGTA |
| OASL | CTGATGCAGGAACTGTATAGCAC | CACAGCGTCTAGCACCTCTT |
| KAL1 | CCTGCAAGGAATCAGGGGAC | GTCAAGCATTCGTAGCTCTTCT |
| MX1 | AGCGGGATCGTGACCAGAT | TGACCTTGCCTCTCCACTTATC |
| GPR84 | TTGGCATCTTCTATTGCCTCATC | TGTCGCAACTTGTATTGGTCC |
| USP18 | AACGTGCCCTTGTTTGTCCAA | GAGTCCTTCACCCGGATCGTA |
| IFIT1 | GCGCTGGGTATGCGATCTC | CAGCCTGCCTTAGGGGAAG |
| IFI44L | AGCCGTCAGGGATGTACTATAAC | AGGGAATCATTTGGCTCTGTAGA |

**Supplemental Table 2:** Steps for Pathway Constrained PCA LASSO Predictor**.**

1. Screen genes univariately, dropping genes with nominal Wilcoxon p-value greater than 0.10.
2. Standardize genes to have mean 0 and SD 1 prior to forming pathway principal components (PC).
3. Create pathway principal components (PC) using the standardized screened genes.
4. Set to zero (hard-threshold) any loadings whose |loading| < 0.75*mean(|loadings|) within that pathway.
5. Screen the pathway first hard-thresholded PC (HTPC) using a Bonferroni Wilcoxon p-value (with alpha selected by CV).
6. Standardize each pathway first HTPC to have mean 0 and SD 1 before using LASSO to estimate pathway OR.
7. Perform LASSO on the screened pathway first HTPC, selecting the LASSO penalty via CV (along with the screening level).
8. If any LASSO OR < 1, take the reciprocal (so OR >= 1) and swap the signs of all of the loadings for that pathway.
9. Shrink to one (hard-threshold) any LASSO OR < $\text{mean(OR)}^{0.75}$

**Supplemental Table 3:** Pathogens Identified.

| Pathogen | Number of detections |
|---|---|
| **Viruses** | 68 |
| Adenovirus | 1 |
| Coronaviruses | 12* |
| Influenza A | 22 |
| Influenza B | 4 |
| Human metapneumovirus | 11* |
| Parainfluenza viruses | 2 |
| Rhinoviruses | 9 |
| Respiratory Syncytial virus | 7 |
| **Bacteria** | 44 |
| *Streptococcus pneumoniae* | 14 |
| *Haemophilus pneumoniae*† | 9 |
| *Staphylococcus aureus* | 6 |
| *Moraxella catarrhalis* | 3 |
| Beta hemolytic streptococci | 5 |
| *Chlamydophila pneumoniae* | 3 |
| *Legionella pneumophila*† | 2 |
| Coagulase negative staphylococcus‡ | 1 |
| Streptococcus salivarius‡ | 1 |

*****One subject had mixed HMPV and coronavirus

**†** One subject had mixed Legionella and *Haemophilus influenzae*

**‡** Multiple blood cultures positive, source unclear

An additional 33 subjects had viruses and bacteria identified by did not meet predefined criteria to reliably classify subjects as viral, bacterial or mixed infection. These subjects were predominately those with viral infection without adequate studies to “rule out” concomitant bacterial infection.

FilmArray Respiratory Panel, Idaho Technologies, Inc, Salt Lake City, UT included: Adenovirus, Coronavirus (CoV) HKU1, CoV NL63, CoV 229E, CoV OC43, Human metapneumovirus (HMPV), Influenza A, (H1, H3, and subtype 2009 H1), Influenza B, Parainfluenza Viruses 1-4, Rhinovirus/Enterovirus, RSV, *Chlamydophila pneumoniae*, *Mycoplasma pneumoniae*, and *Bordetella pertussis*.

**Supplemental Table 4: Thresholds for the nominal predicted probability of a bacterial infection**

| Cutoff | **Naïve Results** | | | | **Nested Cross-Validated** | |
|---|---|---|---|---|---|---|
| | Sensitivity | Specificity | # Bacterial Classified as Not Bacterial | # Not Bacterial Classified as Bacterial | Sensitivity | Specificity |
| 0.35 | 0.951 | 0.679 | 2 | 17 | 0.854 | 0.670 |
| **0.40** | **0.902** | **0.830** | **4** | **9** | **0.793** | **0.759** |
| 0.45 | 0.805 | 0.887 | 8 | 6 | 0.744 | 0.816 |
| 0.50 | 0.707 | 0.962 | 12 | 2 | 0.665 | 0.868 |

**Supplemental Table 5: Clinical Characteristics of Misclassified Subjects.**

| | Subject ID | Age | Nasal Congest | Sputum | Wheeze | Rigors | Temp ®C | SBP | WBC | Anion Gap | BUN | PCT | CXR Infiltrate | Microbiologic Diagnosis | Illness Class | Clinical Diagnosis |
|---|---|---|---|---|---|---|---|---|---|---|---|---|---|---|---|---|
| Bacterial as non-bacterial | | | | | | | | | | | | | | | | |
| | N0D129 | 50 | No | Yes | Yes | No | 37.0 | 105 | 11.3 | 12 | 11 | 0.05 | No | *H. influenzae* | Bacterial | Bronchitis |
| | NAB06D | 50 | Yes | Yes | Yes | No | 38.1 | 131 | 7.0 | 9 | 14 | 0.05 | No | Influenza A/ Moraxella | Viral Bacterial | Bronchitis |
| | NAB3B7 | 73 | Yes | Yes | No | No | 37.4 | 102 | 16.8 | 9 | 16 | NA | Yes | Influenza A/ *S. aureus* | Viral Bacterial | Bacteremic Pneumonia |
| | NC08A0 | 70 | No | Yes | No | No | 37.4 | 109 | 15.9 | 9 | 10 | NA | Yes | Group A Streptococcus | Bacterial | Pneumonia |
| Non-bacterial as bacterial | | | | | | | | | | | | | | | | |
| | N94C54 | 72 | Yes | Yes | No | No | 37.5 | 92 | 5.9 | 8 | 10 | 0.37 | No | Rhinovirus | Viral | COPD |
| | N7BFA3 | 41 | Yes | Yes | Yes | Yes | 39.1 | 90 | 15.8 | 9 | 12 | NA | Yes | HMPV | Viral | Pneumonia |
| | N7F9AA | 61 | Yes | Yes | No | Yes | 39.5 | 104 | 5.3 | 7 | 12 | 0.12 | No | Influenza A | Viral | Bronchitis |
| | NB3035 | 49 | No | Yes | Yes | No | 38.3 | 94 | 7.4 | 14 | 14 | 0.06 | No | HMPV | Viral | Bronchitis |
| | N8323B | 80 | No | Yes | Yes | No | 37.3 | 138 | 12.1 | 12 | 31 | 0.31 | No | RSV | Viral | Asthma |
| | NF73CC | 82 | No | No | No | No | 39.0 | 101 | 9.6 | 10 | 15 | 0.05 | No | Coronavirus | Viral | Bronchitis |
| | NA240D | 66 | Yes | Yes | No | No | 36.2 | 98 | 7.8 | 9 | 16 | NA | No | Influenza B | Viral | COPD |
| | NA8BE9 | 77 | No | Yes | Yes | Yes | 37.3 | 115 | 11.6 | 8 | 20 | NA | No | HMPV | Viral | Bronchitis |
| | N45520 | 51 | No | Yes | Yes | No | 36.7 | 133 | 13.2 | 8 | 9 | NA | No | HMPV | Viral | COPD |

SBP=Systolic blood pressure (mm Hg), WBC=White blood cell 1000 cells/mL, BUN = Blood urea nitrogen (mg/dL), PCT= Procalcitonin ng/dL), CXR=Chest radiograph, HMPV=human metapneumovirus, RSV= respiratory syncytial virus

## Supplemental Table 5: Footnote Clinical Vignettes

**N0D129** Patient history of asthma/COPD, presented with 3-4 days of wheezing, dyspnea and increased thick yellow phlegm. Good quality sputum grew 3+ H. influenzae. Treated with antibiotics and recovered.

**NAB06D** Patient with history of asthma/COPD, recent sick contacts, presented with 2 days of fever, dyspnea and productive cough. CXR was without infiltrate. Patient tested positive for influenza A and good quality sputum grew 4+ M. catarrhalis. Patient was treated with oseltamivir and antibiotics and recovered.

**NAB3B7** Detailed history provided in Figure legend for Figure S4

**NC08A0** Patient presented with severe sore throat, fever, body aches, dizziness, cough and confusion. Rapid strep test was positive for Group A streptococcus (GAS). CXR showed a patchy infiltrate in the right lung base. Sputum after antibiotics grew 1+ GAS. Patient received antibiotics and recovered.

**N94C54** Patient with end stage renal disease, COPD and CHF presented with 2 days of dry cough, sore throat and dyspnea. Felt to have primarily CHF exacerbation. No antibiotics given.

**N7BFA3** Patient with asthma presented with 1 day of nasal congestion, fever, dyspnea and wheezing with green sputum production. Husband sick with similar illness. Patient had patchy infiltrate in the right lung base and good quality sputum that grew only normal respiratory flora and blood cultures were negative. She was treated with antibiotics for community acquired pneumonia and recovered.

**N7F9AA** Patient present presented with 1 day of scratchy throat, dry cough, dyspnea with fever and myalgia. CXR was without infiltrate. Patient was tested positive for influenza A and was treated with oseltamivir without antibiotics and recovered.

**NB3035** Patient with coronary artery disease, CHF and tobacco and alcohol abuse presented with nasal congestion for several days followed by increasing dyspnea with cough productive of foamy blood streaked sputum. CXR showed edema and sputum culture was negative for pathogens. Impression from the cardiologist was viral bronchitis induced CHF. The hospitalist was concerned for pneumonia and the patient received antibiotics and recovered.

**N8323B** Patient with asthma presented with 2 days of nasal congestion, hoarseness, fever, wheezing and yellow sputum. CXR was negative for infiltrate and sputum grew normal flora. She received antibiotics and recovered.

**NF73CC** Patient with CHF presented with several days of nasal congestion, sore throat, fevers and then developed a cough productive of yellow sputum. CXR showed no infiltrate, sputum grew normal flora. Antibiotics were given initially then stopped due to low suspicion of bacterial infection and he recovered

**NA240D** Patient with history of heroin abuse and COPD presented with headache, body aches, chills and dyspnea. CXR showed basilar atelectasis. Patient tested positive for influenza and was treated with oseltamivir. Antibiotics were given initially then stopped due to low suspicion of bacterial infection and she recovered.

**NA8BE9** Patient with asthma presented with 4 days of chills, wheezing and cough. CXR was negative and sputum grew normal flora but patient received antibiotics and recovered.

**N45520** Patient with asthma presented with 3 days of wheezing, dyspnea and clear sputum. CXR was clear, no antibiotics were given and patient recovered

**Figure S1**

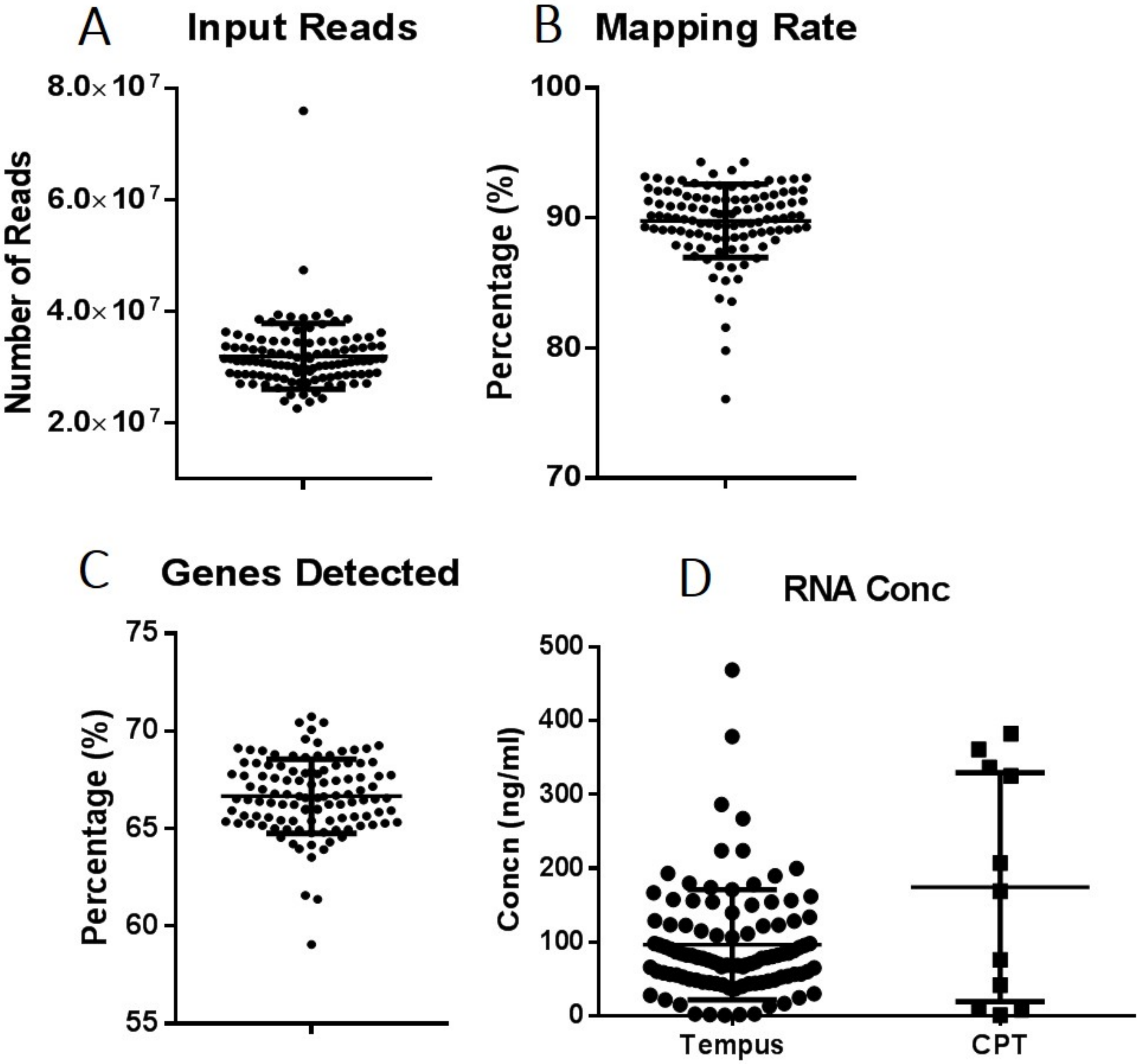

A
Input Reads
8.0×10⁷
6.0×10⁷
4.0×10⁷
2.0×10⁷
Number of Reads
B
Mapping Rate
100
90
80
70
Percentage (%)
C
Genes Detected
75
70
65
60
55
Percentage (%)
D
RNA Conc
500
400
300
200
100
0
Concn (ng/ml)
Tempus
CPT

**Supplemental Figure S2**

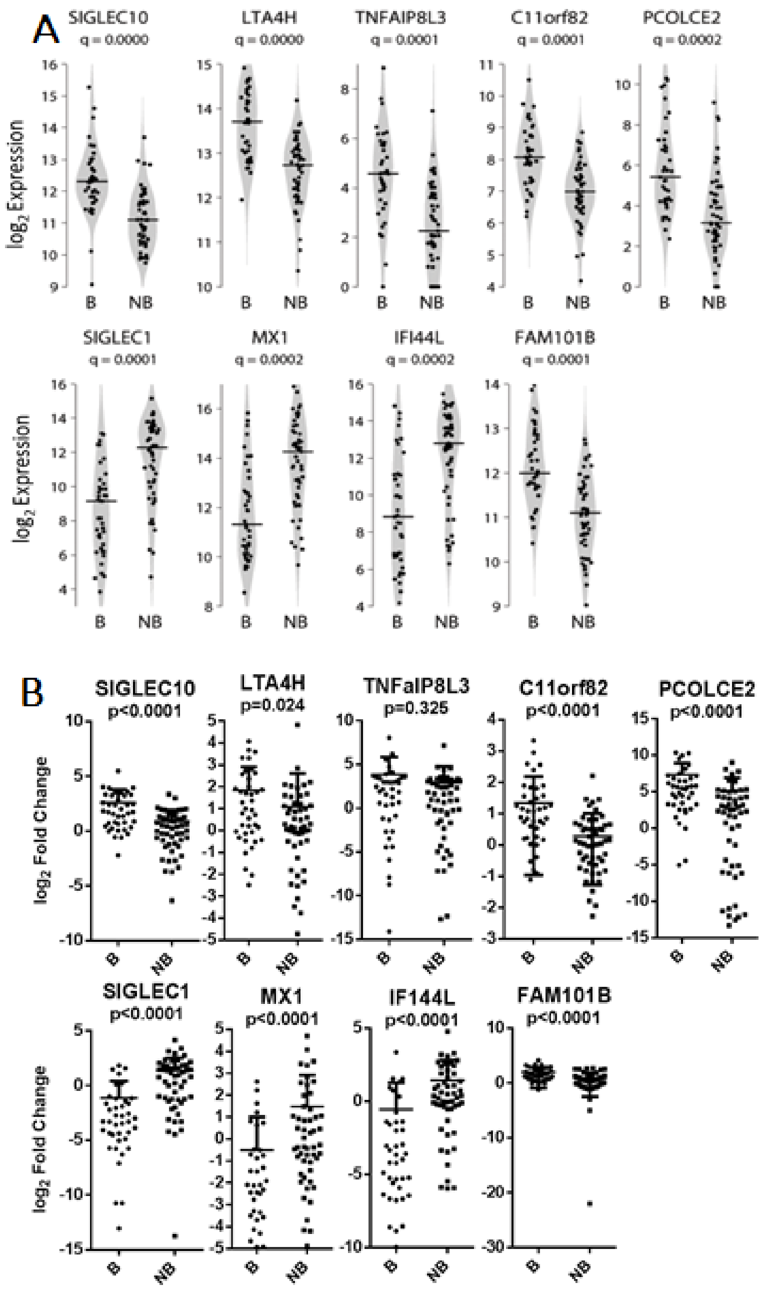

## Supplemental Figure S3

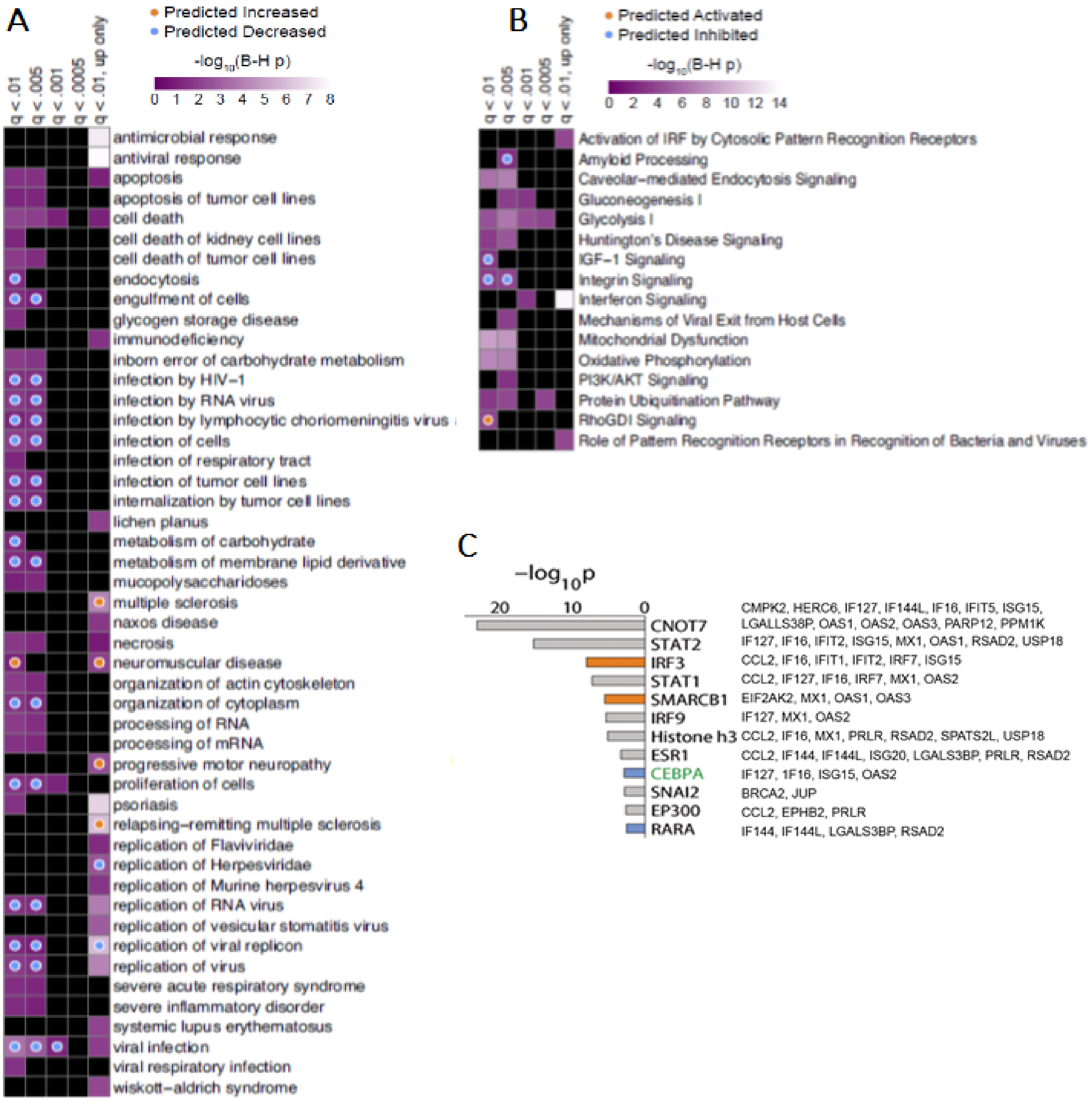

**Supplemental Figure S4**

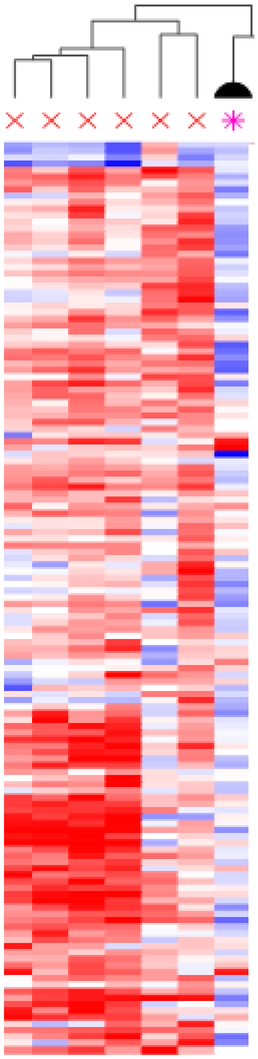

Supplement: Supplementary file 1 — Supplementary Information [file 41598_2017_6738_MOESM1_ESM.pdf]
